# Rett syndrome from bench to bedside: recent advances

**DOI:** 10.12688/f1000research.14056.1

**Published:** 2018-03-26

**Authors:** Yann Ehinger, Valerie Matagne, Laurent Villard, Jean-Christophe Roux

**Affiliations:** 1 Aix Marseille Univ, INSERM, MMG, 13385 Marseille, France

**Keywords:** Rett syndrome, Mecp2, treatment

## Abstract

Rett Syndrome is a severe neurological disorder mainly due to *de novo* mutations in the methyl-CpG-binding protein 2 gene ( *MECP2*). Mecp2 is known to play a role in chromatin organization and transcriptional regulation. In this review, we report the latest advances on the molecular function of Mecp2 and the new animal and cellular models developed to better study Rett syndrome. Finally, we present the latest innovative therapeutic approaches, ranging from classical pharmacology to correct symptoms to more innovative approaches intended to cure the pathology.

## Introduction

Rett syndrome (RTT) is a severe neurological disorder with an incidence of 1 in about 15,000, which accounts for up to 10% of severe intellectual disability of genetic origin in women ^1^. The clinical course of the disease consists of an initial normal development until 6 to 18 months of age followed by an arrest of brain development, severely impaired expressive language, the development of stereotypic hand movements, and the appearance of gait ataxia and truncal apraxia/ataxia between 1 and 4 years of age. Other frequent symptoms include breathing dysfunction, electroencephalography (EEG) abnormalities, seizures, spasticity, scoliosis, and reduced growth ^2, 3^. RTT is caused mainly by *de novo* mutations in the methyl-CpG-binding protein 2 ( *MECP2*) gene ^4^.

## MECP2-pathies

In addition to RTT, *MECP2* mutations have been identified in individuals with syndromes such as mild learning disability in females, neonatal encephalopathy in males, and psychiatric disorders, autism spectrum disorders, and X-linked intellectual disability in both males and females ^5^. Interestingly, duplication or triplication of the Xq28 region containing the *MECP2* gene results in a postnatal phenotype, known as the *MECP2* duplication syndrome, in boys ^6^. In most cases, the *MECP2* duplication is inherited from the mother, who expresses a mild to asymptomatic phenotype due to highly skewed X chromosome inactivation (XCI). The main characteristics of this syndrome include early infantile hypotonia, delayed psychomotor development resulting in severe intellectual disability, absent or very limited speech, abnormal gait, epilepsy, and spasticity ^7^. The finding that MECP2 underexpression or overexpression leads to RTT-like phenotypes raises more than one question: is the severity of MECP2-pathies dependent on *MECP2* gene dosage? Are therapeutic projects directed at increasing MECP2 levels in patients considered carefully?

## Cell-specific expression of Mecp2

Mecp2 is widely expressed throughout the body and the highest abundance is in postmitotic neurons, where it contributes to the development and maintenance of synapses ^8^. Moreover, neuron-specific Mecp2 deficiency is sufficient to cause neuronal dysfunction with symptomatic manifestations mimicking the RTT phenotype ^9^. Since these first findings, the idea that Mecp2 expression was only neuronal has evolved, as Mecp2 was reported in non-neuronal central nervous system (CNS) cells, including astrocytes, microglia, and oligodendrocytes, and cell-specific *Mecp2* deletion in these cell subtypes appears to contribute to RTT neuropathology ^10, 11^.

Astrocytes were the first non-neuronal cells reported to express Mecp2 and to play a key role in neuronal morphology ^10, 12^ and RTT symptom progression ^13^. Astrocytes lacking Mecp2 exhibit abnormal features. For instance, microtubule-dependent vesicle transport is altered in Mecp2-deficient astrocytes from Mecp2-deficient mice ^14^. In addition, recent evidence suggests a role of mutant astrocytes in breathing deficits due to a lower chemosensitivity ^15, 16^. Mutant astrocytes were also very recently involved in an abnormal regulation of excitatory synaptic signaling in Mecp2-deficient mice ^17^.

A role for microglia in RTT neuropathology was also reported by Derecki *et al*. ^18^. Their findings were very interesting, as they showed that grafting wild-type myeloid cells in irradiated *Mecp2*-null mice led to repopulation of the brain parenchyma by microglia-like myeloid cells and a reduction in the vast majority of disease-associated abnormalities ^18^. Unfortunately, subsequent studies failed to reproduce these findings ^19^, and, although Mecp2 deregulation in microglia was shown to affect other immune functions ^20, 21^, the mechanisms explaining how microglia might contribute to these modifications remain mostly unknown. One possible explanation is that microglia contributes to end stages of the disease by dismantling neural circuits rendered vulnerable by the lack of Mecp2 ^22^.

Oligodendrocytes were also involved in RTT neuropathology, as the expression of Mecp2 specifically in oligodendrocytes alleviated some of the RTT phenotype ^23^. The level of some myelin-related proteins was also deregulated in the brain of *Mecp2*-null mice ^23^, and Mecp2 was reported to regulate the expression of myelin genes in rat oligodendrocytes ^24^. Altogether, Mecp2 is currently known to play a role in most cell types of the CNS.

## Mecp2 binding and transcriptional regulation

Mecp2 is a member of the methyl-CpG-binding domain (MBD) family of proteins ^25– 27^ that are known to play a role in chromatin organization and transcriptional regulation through binding to methylated CpG sites or 5-hydroxymethylcytosine ^28, 29^. Since then, Guo *et al*. ^30^ have shown that Mecp2 was also able to bind to methylated CpA ^30^, and Sugino *et al*. ^31^ showed that Mecp2 repression was biased toward long genes. In 2015, the Greenberg lab revealed that that bias was achieved through binding to mCAs that were more frequent within these long genes ^32^. Recently, the third base following mCA was shown to strongly affect Mecp2 binding, and the strongest binding was the tri-nucleotide sequence mCAC ^33^. Further studies will be necessary to determine whether Mecp2 binding to either mCG or mCAC leads to different biological responses.

Mecp2 was found to be expressed in mouse embryonic development from E10.5 ^8^. Stroud *et al*. recently showed that a DNA methyltransferase (DNMT3A) binds to lowly expressed genes in embryonic stages leading to CA methylation, which subsequently recruits Mecp2 and enables gene repression in the maturing brain ^34^.

In addition to directly binding to methylated DNA, Mecp2 was found to regulate gene transcription through the recruitment of corepressors such as the NCoR/SMRT corepressor complex ^35^. Using a highly truncated version of Mecp2 retaining the MBD domain, the NLS, and the NCoR/SMRT signals, the team of Adrian Bird demonstrated that almost all functions of the Mecp2 protein were maintained, definitively demonstrating the leading role of this complex in the pathology ^36^.

Finally, Mecp2 directly modulates chromatin architecture via its three AT-hook-like domains ^37^. AT-hooks are short DNA-binding motifs that interact with the wide minor groove of AT-rich DNA regions. A study proposed that AT-hooks function as secondary DNA-binding domains either stabilizing or modulating the association of Mecp2 to chromatin. The authors also state that this secondary role is supported by the absence of RTT-causing missense mutations at these AT-hooks sites ^38^. This view may be challenged by recent findings showing that a mouse carrying an eight-amino-acid deletion in the AT-hook domain 1 exhibited locomotor and cognitive dysfunctions ^39^.

These recent findings highlight the complexity of mechanisms by which Mecp2 plays its role of transcriptional regulator and partly explains why therapies other than those aimed at correcting/replacing Mecp2 are able to improve only some of the symptoms in patients with RTT (see below).

## Preclinical models of Rett syndrome

Several RTT mouse models have been developed and provided scientists with invaluable tools to understand the neurobiological mechanisms underlying RTT. Over the past three years, thanks to new gene-editing methods such as the transcription activator-like effector nucleases (TALENs), zinc finger nuclease technology, and the CRISPR-Cas9 system, models in species other than mice have been developed ^40^.

In 2016, two novel *Mecp2*-knockout rat models have been developed by using zinc finger nuclease technology. In the first one, rats present a deletion of exon 4, leading to the complete absence of the Mecp2 protein ^41^. *Mecp2*-null rats recapitulate general RTT features such as growth retardation, malocclusion, hypoactivity, and early death. Null rats exhibit weaker forelimb grip strength and reduced locomotion activity. Breathing abnormalities such as hyperventilation and high apnea rate were found, and this is similar to what is seen in RTT patients and mice. In the second model ^42^, a *Mecp2*-knockout rat was generated and here too the rats presented a symptom progression close to that of the RTT mice. Male rats showed an advanced disease time course and a shorter lifespan compared with female rats. Female Mecp2-deficient rats also recapitulated the motor and behavioral phenotype observed in existing mouse models. More recently, two studies published by the Smith lab showed neuronal cytoskeletal gene dysregulation and mechanical hypersensitivity in the male rat model and non-cognitive deficits (motor, somatosensory, viscerosensory, and metabolic impairments) in the female rat model ^43, 44^. To date, research using these new rat models has confirmed the observations made in the mouse; it will be interesting to see whether future studies will only replicate and validate results obtained in the mouse or bring new findings specific to the rat.

In 2014, the first non-human primate carrying a *MECP2* mutation using TALEN-mediated gene targeting was generated ^45^. The authors obtained one male cynomolgus monkey with a *MECP2* deletion out of the 51 TALEN-injected embryos. They suggest that the low pregnancy and survival rates could be due to TALEN toxicity or that the TALEN-mediated deletion in males could be lethal, such as in the human disease. A mosaicism of *MECP2* deletion was found in the monkey and could be due to a delayed TALEN targeting during embryonic development. The monkey failed to survive, but these results demonstrated the feasibility of *MECP2* editing in non-human primates. In 2017, the same group published a study reporting five living *MECP2*-mutant females ^46^. Magnetic resonance imaging scanning showed significantly reduced cortical gray matter volumes in mutant monkey brains. In addition, mutant monkeys displayed behavioral features such as fragmented sleep, increased stereotypy, and reduced environmental exploration, but no differences in body weight and head circumference were observed. This study seems to validate a robust female monkey model displaying RTT features—such as male embryonic lethality, social withdrawal, and eye-tracking defect—absent in rodent models. This model will be invaluable to investigate therapies, such as gene therapy, that need to be transferred to the clinics and may be impacted by the species barrier. However, given the slower development in the monkey, their longer lifespan, and the cost involved in caring for and maintaining their well-being, it is quite obvious that smaller animal models (rodents and zebra fish) will be the first ones to be used in RTT mechanisms and therapy screening studies.

## *In vitro* models of Rett syndrome

Besides animal models, the use of neuronal cultures has facilitated the understanding of Mecp2’s molecular functions. However, until recently, only rodent primary neurons and human immortalized cell lines were available. Thanks to the forced reprogramming of human somatic cells into induced pluripotent stem cells (iPSCs) ^47^, human neurons (or any other cell type) can be generated from cells of patients with RTT and be used to study RTT. Using human iPSCs, a recent study showed that the lack of MECP2 led to the de-repression of genes on the inactive X chromosome and to transcriptional deregulations of mitochondrial membrane proteins ^48^. The use of astrocytes differentiated from RTT human iPSCs showed a profound transcriptional deregulation in MECP2-deficient cells ^49^ and confirmed that astrocyte-conditioned medium had adverse effects on the cellular physiology and morphology of wild-type neurons ^12^. Using neurons differentiated from MECP2-deficient iPSCs, another study showed a significant deficit in KCC2 expression, a key factor for chloride neuronal homeostasis. These cells consequently have a delayed GABA functional switch from excitation to inhibition. Interestingly, overexpression of KCC2 in these MECP2-deficient neurons rescued GABA functional deficits, suggesting an important role of KCC2 in RTT neuropathology ^50^. In addition to confirming previously described abnormalities in patients with RTT or in mouse models, iPSC deficits can partially be rescued by insulin-like growth factor 1 (IGF-1) treatment ^12^, as was previously shown in an RTT mouse model ^51^. Overall, iPSCs seem to display cellular phenotypes similar to those seen in RTT mouse models and should prove useful in high-throughput screening of new therapeutic compounds.

## Therapeutic approaches

### Gene therapy

As previously mentioned, RTT was shown to be fully reversible in a mouse model of the disease ^52^, indicating that it could be amenable to gene therapy. This is of primary importance, as most RTT mutations occur *de novo* and the disease is usually diagnosed when the symptoms are present, which also means that any therapeutic intervention will be administered to RTT patients after disease onset. Moreover, in order to be efficient, any gene therapy vector will have to reach the whole CNS, as it is globally affected in RTT. Gadalla *et al*. showed, as a proof of principle, a considerable improvement when neonatal RTT male mice were administered a gene therapy vector expressing the human MECP2e1 isoform by intracranial delivery ^53^. The phenotypic rescue was less pronounced when a more translational approach (that is, systemic administration of the therapeutic vector in juvenile RTT mice) was used. This first publication was followed by a second one reporting similar results in male RTT mice as well as a phenotypic improvement in female RTT mice ^54^. More recently, our lab has shown that using a codon-optimized Mecp2e1 isoform improved RTT symptoms, including breathing defects, which had not been shown before ^55^. Although these results seemed very promising, some severe side effects have been reported after the administration of high doses of therapeutic vector, which has led to the development of second-generation therapeutic vectors devoid of side effects but with decreased therapeutic efficacy ^56^. Another study, focusing on the route of administration, showed that this second-generation vector had better efficacy when administered via an intra-cerebrospinal fluid route ^57^. Recently, the neonatal intracranial administration of a truncated Mecp2 was also shown to partially rescue the RTT phenotype ^36^, which shows that gene therapy can still be improved and benefit from new discoveries related to Mecp2 biology. Expressing Mecp2 in cases of loss-of-function mutation seems the best option, but what about cases in which a missense mutation leads to the expression of a non-functional Mecp2 protein? Will it act in a dominant-negative manner and prevent a healthy Mecp2 from manifesting its rescuing effect? A study by Gadalla *et al*. indicates that gene therapy would also work in the case of certain missense mutations, since the therapeutic vector was able to improve the RTT symptoms in a knock-in RTT mouse model (T158M) ^56^. Another therapeutic approach would be to directly correct the missense mutation by gene editing, as was done for a *MECP2* duplication with the CRISPR-Cas9 system ^58^, or by RNA editing using the natural editing capability of the adenosine deaminases acting on RNA (ADAR) to correct G>A mutations ^59^. However, these techniques are still in their infancy and so far their efficacy has been shown only *in vitro*.

### X chromosome reactivation

In female cells, one X chromosome is randomly inactivated and this ensures the same expression of X-linked genes in both male and female cells. Therefore, in female patients with RTT, about half the cells express the mutant version of MECP2 while the other half expresses a normal MECP2 protein. Reactivation of the inactivated X chromosome (Xi), or at least of the (normal) inactivated MECP2 allele, could potentially cure RTT. In order to identify molecules involved in Xi reactivation, a short hairpin RNA (shRNA) library was used in two different studies ^60, 61^ and led to the discovery of factors modulating XCI, including PDPK1 (3-phosphoinositide-dependent protein kinase 1) and AKA (Aurora kinase A), whose *in vitro* pharmacological inhibition was able to reactivate the Xi ^60^. In addition to those factors common to the two studies, Sripathy *et al*. reported that the BMP/TGFb pathway was strongly involved in XCI both *in vitro* and *in vivo* ^61^. In another study, by combining a gene knockdown strategy using antisense oligonucleotides against Xist, one of the key XCI regulators, and a pharmacological approach, Carrette *et al*. demonstrated a synergistic effect on Xi reactivation *in vitro* and *in vivo* ^62^. These studies indicate that Xi reactivation is possible; however, the challenge now will be to translate these results into safe therapeutic interventions. The main issue will be to determine the impact of the induced global X-linked protein overdose following Xi reactivation.

## Clinical trials

Since 1966 and the first clinical description of RTT, over 25 clinical trials testing therapeutic agents targeting motor, cognitive, and autonomous dysfunctions have been initiated. For instance, breathing abnormalities such as apneas, which are crucial in RTT, have been targeted by several molecules, including desipramine ^37^, sarizotan ^63^ (ClinicalTrials.gov identifier NCT02790034), and ketamine ^63, 64^ (ClinicalTrials.gov identifier NCT02562820). Desipramine, an inhibitor of noradrenaline reuptake, was successfully tested in a preclinical study, reducing apneas and rescuing breathing anomalies in a mouse model of RTT, probably by increasing the number of tyrosine hydroxylase (TH) neurons in the brainstem ^65^. The recent desipramine clinical trial did not reveal a clinical improvement in all patients with RTT, but the authors did find an inverse correlation between desipramine concentration and the number of apneas ^37^. Mirtazapine, a desipramine acting-like molecule without its side effects, showed promising preclinical results in RTT mice and could be the next drug of interest ^66^. On the basis of translational studies ^64, 67^, patients are currently being recruited for the sarizotan clinical trial and the one evaluating the efficacy of ketamine is under way.

Brain-derived neurotrophic factor (BDNF) is an important neurotrophic factor playing a key role in RTT; indeed, its deregulation seems strongly correlated with the reduction of dendritic arborization identified in RTT and its overexpression in *Mecp2*-knockout mice partially rescues their phenotype ^68, 69^. These different points make the BDNF pathway one of the most appealing pathways to target in RTT ^70^. BDNF itself is unable to cross the blood-brain barrier (BBB) and needs to be indirectly activated. Fingolimod is able to increase BDNF expression and improved locomotor activity, sensorimotor coordination, and lifespan in RTT mice ^71^, and a clinical trial (ClinicalTrials.gov identifier NCT02061137) is under way. Glatiramer acetate, another BDNF secretion inducer, was first tested on a small cohort of patients with RTT, and an improvement of gait velocity was reported ^72^. A second clinical trial had to be stopped because of a severe adverse effect on patients ^73^. Unlike BDNF, IGF-1 crosses the BBB and activates similar intracellular pathways. Two preclinical studies in RTT mice reported the positive effect of IGF-1 ^50, 74^. The IGF-1 full-length, also named mecasermin, was tested in three clinical studies ^75– 77^. A total of 10 patients with RTT treated in a preliminary 20-week open-label assessment with mecasermin showed a reduction in the incidence of apneas and improvements of deleterious neurological consequences, including depression and anxiety ^75^. However, a recent placebo-controlled crossover clinical trial on 30 patients with RTT did not succeed in confirming the improvements observed in the previous study ^77^. After a pilot study investigating the safety of the IGF-1 tripeptide form ^78^, a clinical trial with trofinetide (an analogue of the IGF-1 tripeptide) was recently published and presented improvements in core features of RTT ^79^.

A disruption in the balance between excitation and inhibition and between GABA and glutamate pathways is implicated in RTT ^80^. Dextromethorphan, an NMDA receptor antagonist, has been tested for its capacity to restore normal EEG function and reduce seizure, and although no significant improvement in global severity was noticed, statistically significant changes were seen in clinical seizures, language, and behavioral hyperactivity ^81^.

Recent studies have suggested a systemic redox imbalance in a mouse model and in patients with RTT ^82– 84^. Polyunsaturated fatty acids (PUFAs) are US Food and Drug Administration-approved oils that act indirectly on this cellular redox imbalance. In two clinical trials, PUFA dietary supplementation was able to reduce oxidative stress markers and improve the biventricular myocardial systolic function ^85, 86^.

The abovementioned clinical trials all originated from preclinical studies that identified promising therapeutic molecules. In most cases, and as seen in preclinical studies, these treatments improved some RTT symptoms. However, one cannot help noticing that these therapeutic benefits are restricted to a (sometimes small) subset of symptoms. These last few years have been marked by a large improvement in innovative therapeutic strategies, such as gene therapy and gene editing. These new approaches are being applied to the RTT field and have already been the subject of a few publications. Unlike pharmacological approaches, these techniques are aimed at curing the disease rather than alleviating RTT symptoms, raising great hope for patients with RTT and their families. However, these first publications also highlighted the need for increased safety, given the irrevocable nature of the proposed treatments.

## Conclusions

Since the gene responsible for RTT was identified almost 20 years ago ^4^, a stupefying number of research projects aimed at understanding the mechanisms underlying this pathology and identifying potential therapeutic targets have been conducted. From these studies have stemmed many of the clinical trials whose results have recently been published. Even though many trials reported improvements, these were disappointingly small and restricted to a few symptoms at a time. This may be explained in part by the complex and often controversial functions of Mecp2 that have been unveiled throughout the years: from simple transcriptional repressor to genome-wide noise dampener and from a simple CpG MBD to a protein-binding multiple nucleotide sequence. The study of Mecp2 biology revealed an unexpected complexity, and this probably explains why Mecp2 replacement therapies such as gene therapy, gene editing, or X chromosome reactivation are now thought to be the best options to dramatically improve RTT or one day cure it.
